# Dispositional Flow and Performance in Brazilian Triathletes

**DOI:** 10.3389/fpsyg.2019.02136

**Published:** 2019-09-20

**Authors:** William Fernando Garcia, Renan Codonhato, Marcus Vinicius Mizoguchi, José Roberto Andrade do Nascimento Junior, Paulo Vitor Suto Aizava, Marcelen Lopes Ribas, Aryelle Malheiros Caruzzo, João Ricardo Nickenig Vissoci, Lenamar Fiorese

**Affiliations:** ^1^Physical Education Department, State University of Maringá, Maringá, Brazil; ^2^Physical Education Department, Federal University of Mato Grosso, Cuiabá, Brazil; ^3^Physical Education Department, Federal University of São Francisco Valley, Petrolina, Brazil; ^4^Duke Emergency Medicine, Duke University Medical Center, Durham, NC, United States; ^5^Duke Global Health Institute, Duke University, Durham, NC, United States

**Keywords:** flow, endurance, triathlon, sport psychology, athletes

## Abstract

Flow is a mental state characterized by total immersion and focus in an activity; performing it pleasurably. Such a state is considered optimal for performance. The present study analyzed the relationship between dispositional flow and performance in triathletes. The sample consisted of 328 athletes (294 males and 34 females; mean age of 37.42 ± 7.18 years) competing in the Ironman Brazil – Florianópolis – South American Championship 2017. Instruments were an identification sheet, the Dispositional Flow Scale (DFS-2) and athletes’ total race times. Data were analyzed using R, through the Shapiro–Wilk normality test, Mann–Whitney’s *U*, Spearman Correlation, and Network Analysis [Least Absolute Shrinkage and Selection Operator (LASSO)], using strength, closeness, and betweenness as centrality measurements. Results show a positive correlation between age and practice time (*r* = 0.34), inverse relationship between practice time and total race time (*r* = −0.25), and inverse correlations between race time and 05 of the 09 flow dimensions (*r* between −0.17 and −0.11), suggesting better performances were related to more practice time and higher disposition to flow. Flow conditions, flow characteristics, individual characteristics, and performance were separately grouped in the network structure. Challenge–skill balance was the most influential node, with the highest closeness and betweenness values; challenge–skill balance, clear goals, control, and action-awareness merge directly influenced better race times. Sample’s top 50 performers had significantly higher disposition to challenge-skill balance, clear goals, control and feedback. Practical implications of flow mechanisms are discussed. Dispositional flow was positively related to objective performance in Brazilian triathletes.

## Introduction

Sports psychology strives to promote better performance to coaches and athletes within their sport’s context (Weinberg and Gould, 2017). In that sense, the positive psychology approach has gained importance among researchers and professionals involved with sports and sports psychology because it seeks to understand and promote positive aspects of the human mind, recognizes the importance of subjective experiences and well-being, and aims to go further than just repairing damage (Seligman and Csikszentmihalyi, 2000). Among the investigated aspects of positive psychology, flow state has been highlighted as a key aspect for studying and understanding athletes’ peak performance (Swann et al., 2017). Flow state is a harmonious, highly positive, pleasurable, and intrinsically rewarding psychological state, characterized by intense focus, deep absorption in an activity, and a sense of things “clicking into place,” despite any challenges (Csikszentmihalyi, 2002).

Flow theory (Csikszentmihalyi, 2002) describes the experience of flow in nine dimensions, being six characteristics of such a mental state: (1) intense concentration; (2) merging of awareness (what is perceived) and action (how to act/react); (3) decreased awareness of social evaluation or self-judgment (loss of self-consciousness); (4) sense of control over performing an activity and its outcomes; (5) transformation of time, seeming to either speed up or slow down; (6) the autotelic characteristic of the experience, of being pleasurable and rewarding. The other three dimensions are considered prerequisites for experiencing flow: (I) balance between challenge and skill, where there is a high challenge that the athlete feels capable of overcoming; (II) clear goals, guiding the athletes’ efforts; (III) unambiguous feedback regarding one’s progress toward set goals (Csikszentmihalyi, 2000).

Flow state has been linked to general sports performance and peak performance (Jackson, 1992, 1996; Jackson and Roberts, 1992; Jackson et al., 1998, 2001; Marsh and Jackson, 1999; Stavrou et al., 2007; Bakker et al., 2011; Swann et al., 2016), demonstrating that athletes feel in such a mental state during their best performances, although it is also possible to experience flow without reaching peak performance (Stavrou et al., 2007). Moreover, flow state can be rare and unpredictable (Chavez, 2008). Meanwhile, there are uncertainties regarding how flow is experienced, its exact definition, the overlapping of some of its dimensions, and its causal mechanisms (Swann et al., 2018).

Despite the contributions of flow theory to sport performance research, many studies have considered the athlete’s performance as a condition for flow state (Swann et al., 2012), while its influence on performance as an outcome still requires more convincing evidence (Engeser and Rheinberg, 2008). Studies have reported a positive relationship between flow and a variety of subjective measurements of performance in a wide range of individual and team sports (Jackson and Roberts, 1992; Jackson et al., 1998, 2001; Marsh and Jackson, 1999; Stavrou et al., 2007; Bakker et al., 2011; Soulliard et al., 2019); however, when objective indicators are used, such as race time or finishing position, the evidence is less clear, and only studies with individual sports were found. Two studies found a positive relationship between performance and flow dimensions, more specifically for the autotelic dimension (Stavrou et al., 2007; 07 individual sports) and the clear goals, challenge–skill balance, and action-awareness dimensions (Jackson et al., 2001; 03 individual sports). Still, two studies with marathon runners found no significant relationship between flow and performance (Stoll and Lau, 2005; Schüler and Brunner, 2009), both adopting a global flow dimension.

In this sense, gaps still exist regarding how this psychological state, described as ideal for optimal functioning, can directly affect athletes’ actual performances. To determine when and if flow state occurred during a certain activity is still a methodological challenge (Jackman et al., 2017). It is possible, however, to assess the frequency of flow experiences and its indicators, obtaining a general disposition of an athlete to experience such a mental state when performing (Jackson and Eklund, 2002). Thus, the present investigation aimed to study the relationship between dispositional flow and objective performance of Brazilian triathletes.

## Materials and Methods

### Participants

The target population consisted of 1455 Brazilian triathletes (1266 males and 189 females) competing in the Ironman Brazil – Florianópolis – South American Championship 2017, considered the largest and most important ultra-endurance triathlon in South America. To represent this population, the sample size was calculated for 95% confidence level and 5% confidence interval, resulting in a required sample of 304 athletes.

Subjects were recruited according to the following inclusion criteria: (a) accept voluntary participation by signing an Informed Consent Term; (b) be registered for the 2017 Ironman Brazil; (c) have Brazilian nationality; (d) be 20–59 years of age. Answering the instruments incorrectly, having technical or health-related problems during the race, and not completing the race were adopted as exclusion criteria.

The final sample comprised 328 athletes (294 men and 34 women), with an average age of 37.42 ± 7.18 years, from all regions of Brazil. These athletes completed the event with times between 8 h 06 min and 16 h 51 min (11.49 ± 1.51).

### Instruments

We used an Identification Sheet, which contained name, age, sex, and time practicing triathlon (in years and months), to characterize our subjects. Athletes’ objective performance was assessed through their total race time in the competition.

Dispositional flow was measured by the Dispositional Flow Scale-2 (DFS-2) developed by Jackson and Eklund (2002) and validated to the Brazilian sport context by Gomes (2014). This instrument assesses an athlete’s subjective perception of several flow state indicators. It is composed of 36 items representing the nine dimensions of flow: challenge–skill balance, action/awareness merging, clear goals, unambiguous feedback, intense concentration, control over the task at hand, loss of self-consciousness, transformation of time, and autotelic experience. Each item is answered in a 5-point Likert-type scale varying between 1-Completely disagree and 5-Completely agree. The score for each dimension is obtained through the mean value of its correspondent items, with higher values indicating higher disposition to experience a certain dimension of flow state. Cronbach’s Alpha for each dimension is shown in Table 1.

**TABLE 1:** Correlation matrix of Brazilian athletes’ individual characteristics, disposition to flow, and performance.

	**1**	**2**	**3**	**4**	**5**	**6**	**7**	**8**	**9**	**10**	**11**	**12**
(1) Age (years)	−											
(2) Practice time (years)	0.31^∗∗^	−										
(3) Challenge-skill balance	0.03	0.10	−									
(4) Action/awareness merging	−0.12^∗^	−0.03	0.46^∗∗^	−								
(5) Clear goals	0.03	0.08	0.55^∗∗^	0.42^∗∗^	−							
(6) Unambiguous feedback	0.04	0.09	0.53^∗∗^	0.38^∗∗^	0.65^∗∗^	−						
(7) Concentration on task	0.10	0.09	0.37^∗∗^	0.36^∗∗^	0.54^∗∗^	0.55^∗∗^	−					
(8) Sense of control	0.00	0.07	0.51^∗∗^	0.46^∗∗^	0.56^∗∗^	0.63^∗∗^	0.72^∗∗^	−				
(9) Loss of self-consciousness	0.01	0.03	0.12^∗^	0.22^∗∗^	0.19^∗∗^	0.25^∗∗^	0.24^∗∗^	0.27^∗∗^	−			
(10) Transformation of time	−0.08	−0.01	0.11^∗^	0.26^∗∗^	−0.02	0.04	0.07	0.06	0.24^∗∗^	−		
(11) Autotelic experience	−0.05	0.08	0.35^∗∗^	0.28^∗∗^	0.47^∗∗^	0.36^∗∗^	0.27^∗∗^	0.29^∗∗^	0.11^∗^	0.21^∗∗^	−	
(12) Total race time (hours)	0.32^∗∗^	−0.25^∗∗^	−0.17^∗∗^	−0.13^∗^	−0.14^∗∗^	−0.11^∗^	−0.08	−0.14^∗∗^	0.09	0.05	−0.02	−
α	N.A.	N.A.	0.61	0.75	0.74	0.76	0.81	0.79	0.83	0.68	0.71	N.A.
*x̄*	37.41	6.18	4.01	3.62	4.24	3.95	3.87	3.85	3.78	3.45	4.57	11.49
SD	7.18	5.26	0.52	0.61	0.49	0.52	0.62	0.55	0.85	0.68	0.45	1.51

*N.A., Not applicable; α, Cronbach’s Alpha; *x̄*, mean values; SD, standard deviation. ^∗^*p* < 0.05; ^∗∗^*p* ≤ 0.01.*

### Procedures

The companies providing training and coaching for athletes competing in this event were also contacted to establish previous contact with athletes. Data collection took place during the week of the event prior to the competition. Athletes who were not being advised/trained by any company were contacted in person during the Ironman Expo and the event’s accreditation, in the 3 days that preceded the race. Questionnaires were given by the researchers and answered individually by the athletes.

### Data Analysis

Data were analyzed through descriptive and inferential statistics using the R software v3.5.1. Missing data were imputed through MICE package (Multiple Imputation Chained Equations). Data distribution was verified by the Shapiro–Wilk test, presenting a non-parametrical univariate distribution. Spearman correlation was used to assess the relationship between variables and Mann–Whitney’s *U* was used to compare sample’s top 50 performers with the others. Values were considered significant at *p* < 0.05.

In order to study the complex interaction between study variables, a Network Analysis technique was applied. By using the qgraph package, a Least Absolute Shrinkage and Selection Operator (LASSO) network was produced, which calculates a network of partial correlations between all variables, promoting associations between pairs while controlling for the influence of other variables. Then the LASSO network shrinks trivially small correlations to zero, plotting a network of only the largest associations, removing potentially spurious correlations (Wang et al., 2018). Networks are formed by “nodes” (circles) representing variables and “edges” connecting variables. Edges’ colors indicate the direction of the relationship, and the edges’ width represents the strength of the association. Nodes’ positioning within the network also follows the calculated associations (Silva et al., 2006). In the present network, positive associations were represented by blue edges, while red edges indicated an inverse relationship.

Besides visual inspection of the network, the following centrality indices were used to identify the most influential nodes: strength of the connections; closeness centrality, which measures the distance between nodes and indicates how easily a node’s information travels through the network; and Betweenness centrality, describing the number of times a node acts as a bridge in the shortest path between two nodes, which indicates the node’s potential to affect other variables within the network (Dalege et al., 2017).

## Results

Analysis of the correlation matrix for the investigated variables (Table 1) showed significant positive correlations between athletes’ age and total race time (*r* = 0.32), suggesting better performances for younger athletes. An inverse significant relationship was found for time of practice and race time (*r* = −0.25), indicating better race times for more experienced athletes. Five dimensions of disposition to flow were correlated with better race times: challenge–skill balance (*r* = −0.17), action/awareness merging (*r* = −0.13), clear goals (*r* = −0.14), feedback (*r* = −0.11), and control over task at hand (*r* = −0.14).

**FIGURE 1:**
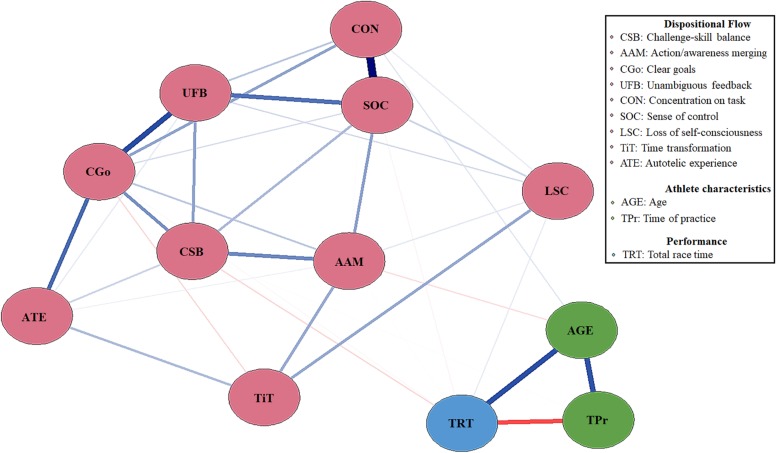
Least Absolute Shrinkage and Selection Operator Correlation Network for athletes’ characteristics, dispositional flow, and performance.

The resulting network (Figure 1) presented a noticeable grouping and separation of variables, with objective measurements (age, hours of practice, and performance) on one side and flow indicators seemingly spread on the other. Dispositional flow dimensions were positively associated with one another, showing a positive interaction between dimensions. The three dimensions representing prerequisites to flow state (challenge–skill balance, clear goals, and feedback) were closely positioned, forming a triangle, which is encompassed by five characteristics of flow state, with only loss of self-consciousness being positioned further to the side. Athletes’ ages presented a positive correlation with intense concentration while being negatively linked to action/awareness merging. Furthermore, the amount of triathlon practice was not related to these athletes’ disposition to experience flow.

Taking a closer look at the interaction between flow dimensions within the network, we observe a strong connection between intense concentration and the sense of control over such activity (*r* = 0.47); such sense of control was also connected to feedback (*r* = 0.25), action/awareness merging (*r* = 0.16), and challenge–skill balance (*r* = 0.12). Together, these four dimensions were positioned in a square-shaped aspect at the core of all nine dimensions. Clear goals (*r* = 0.26) and challenge–skill balance (*r* = 0.07) have contributed to the autotelic characteristic of the experience. Transformation of time was positively linked to action/awareness merging (*r* = 0.15), loss of self-consciousness (*r* = 0.15) and autotelic experience (*r* = 0.12), on the other hand, it has presented a small but negative relationship with clear goals (*r* = −0.05).

Athletes’ total race time has presented a positive relationship with age (*r* = 0.31) and loss of self-consciousness (*r* = 0.03), while practice time was the most relevant indicator of lower race times (*r* = −0.26). Four dimensions of flow have presented weak negative connections with race time, two pre-conditions to flow: challenge–skill balance (*r* = −0.05) and clear goals (*r* = −0.02); and disposition to experience two characteristics of flow: control over the task at hand (*r* = −0.02) and action/awareness merging (*r* = −0.01). Weight of all associations within the network are shown in Table 2.

**TABLE 2:** Weight of associations within the Correlation Network for Brazilian.

	**1**	**2**	**3**	**4**	**5**	**6**	**7**	**8**	**9**	**10**	**11**	**12**
(1) Age (years)	−											
(2) Practice time (years)	0.30	−										
(3) Challenge-skill balance		0.01	−									
(4) Action/awareness merging	–0.05		0.21	−								
(5) Clear goals			0.19	0.09	−							
(6) Unambiguous feedback			0.16		0.31	−						
(7) Concentration on task	0.04			0.01	0.16	0.09	−					
(8) Sense of control			0.12	0.16	0.06	0.25	0.47	−				
(9) Loss of self-consciousness				0.04		0.06	0.03	0.07	−			
(10) Transformation of time				0.15	–0.05				0.15	−		
(11) Autotelic experience			0.07	0.03	0.26	0.04				0.12	−	
(12) Total race time (hours)	0.31	–0.26	–0.05	–0.01	–0.02			–0.02	0.03			−

**FIGURE 2:**
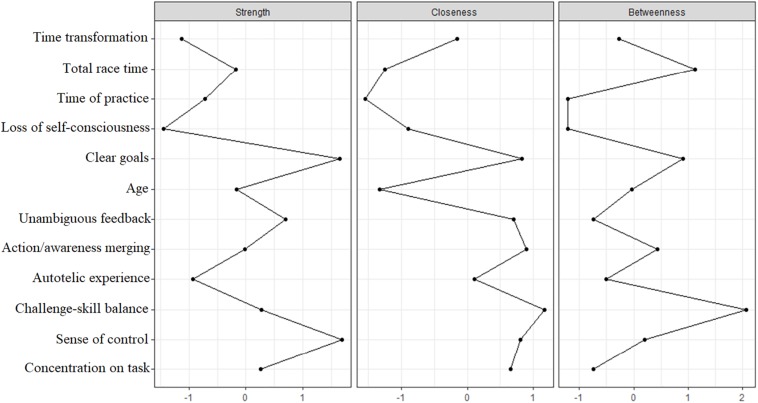
Strength, closeness, and betweenness centrality of network’s variables.

Analyzing the network centrality indices (Figure 2), we observe that clear goals and sense of control were the most strongly connected variables within the network; challenge–skill balance has presented the highest closeness value, followed by action/awareness merging, sense of control, and clear goals; challenge-skill balance had the highest values of betweenness as well, thus highlighting it as the most influential node in the network.

Lastly, as a way of visualizing our results in a simpler manner, the 50 best performers in our sample were grouped and had their data compared with the other 278 subjects’. We found that these top 50 athletes were younger (*p* = 0.01), had practiced triathlon for longer (*p* = 0.02), and had higher levels of dispositional challenge–skill balance (*p* = 0.02), clear goals (*p* = 0.02), unambiguous feedback (*p* = 0.02), and sense of control (*p* = 0.03).

## Discussion

### Flow and Performance

The present investigation aimed to study the relationship between dispositional flow and performance of Brazilian triathletes. Five of the nine flow dimensions were correlated to better race times (Table 1), and four of these correlations remained significant in the LASSO network analysis (Figure 1). Moreover, sample’s top 50 performers presented higher levels of four dimensions of flow when compared to the others. Such results indicate that dispositional flow can be positively related to triathletes’ performance.

Despite the low strength of the associations, which could be attributed to the general characteristic of the DFS-2, an instrument that is not event-specific, present results are still in agreement with existing evidence. Jackson et al. (2001) have reported that clear goals (β = −0.24), challenge–skill balance (β = −0.19), and action/awareness merging (β = −0.15) were predictors of better finishing position in orienteering, surf lifesaving, and cycling athletes (*n* = 236, 66% male). Another study (Stavrou et al., 2007), with athletes from seven different individual sports (*n* = 220, 51% male), reported a positive relationship between objective performance and eight of the nine flow dimensions (*r* values between 0.19 and 0.52); however, only the autotelic experience dimension remained a significant predictor of performance after multiple regression analysis (β = 0.37). Both of these studies have assessed subject’s flow through the Flow State Scale, an event-specific instrument (FSS, Jackson and Eklund, 2002).

Furthermore, two other studies have investigated the relationship between flow and objective performance in marathon runners. Stoll and Lau (2005) reported an inverse non-significant relationship between unidimensional flow and total race time (*r* = −0.11) (*n* = 160, 79% male), while Schüler and Brunner (2009) (*n* = 112, 68% male) have also found an inverse non-significant relationship between unidimensional flow and marathon race time (*r* = −0.12, β = −0.08). Despite presenting the same direction of relationship between flow and performance, these two studies have used not only smaller sample sizes, but they have also measured flow through another instrument, the Flow Short-Scale (Rheinberg et al., 2003), which could explain the lack of statistical significance for the reported relationships.

A few factors must be taken in consideration when comparing these studies. Present results and literature evidence were similar in the sense that only individual sports were studied and samples were predominantly male, with the exception of one (Stavrou et al., 2007). Moreover, all of the reported results suggest that flow will positively influence objectively measured athletic performance. On the other hand, three different instruments were used to assess flow across studies. Furthermore, objective performance was measured in three different ways: race times (present study, Stoll and Lau, 2005; Schüler and Brunner, 2009), finishing position (Jackson et al., 2001), and different calculations accounting for the athletes’ best performance, performance goal, and actual result (Stavrou et al., 2007). These factors, along with differences in sample sizes, could explain the different strengths and significance of associations across studies.

### Flow Mechanisms

The data analysis method adopted here allows for the observation of how variables interact in a mutual and multivariate way. It is a data-driven exploratory method of analysis considered to be adequate to represent complex relationships in the form of a network graph (Wasserman and Faust, 1994). By analyzing the present network (Figure 1), it is possible to suggest explanations for flow mechanisms, looking at how dimensions interact with each other and how these subjective measurements are linked to these athletes’ actual performance.

In this sense, the dimension of challenge–skill balance appeared as the most influential node within the network, being the closest to all other eight dimensions as well as acting as a bridge between them; in other words, changes in this dimension have the highest potential to influence other dimensions of flow and, consequently, their outcomes. Such observation is in accordance with what is proposed by the Flow Theory (Csikszentmihalyi, 2000), which recognizes the importance of having balance between challenge and skill for the experience of flow as a whole. Following this dimension and its connections, we observe a close relationship with clear goals and unambiguous feedback. Together, challenge–skill balance, clear goals, and unambiguous feedback were grouped in the shape of a triangle; coincidently, these are the three dimensions considered to be flow prerequisites (Csikszentmihalyi, 2002).

Encompassing the three flow prerequisites, we observe five characteristics of flow experience positioned between these variables and subjects’ objective measurements, with only the loss of self-consciousness dimension standing further to the side. There are a series of interactions among the nine dimensions of flow. Starting from the strong connection between clear goals and unambiguous feedback, we observe two other interactions stemming from this dyad: (1) the autotelic characteristic of the experience was positioned at the side of clear goals, suggesting the importance of goal setting and achievement for pleasurable and rewarding experiences; (2) the sense of control has been positioned at the side of unambiguous feedback, indicating that it can influence, or be influenced by, progress toward set goals and knowing how to adjust in order to improve.

Athletes’ sense of control has been strongly linked to the concentration on task, which highlights the importance of concentration to achieve control over an activity. Moreover, clear goals were positively related to concentration, while challenge–skill balance presented a positive association with sense of control, thus reinforcing how flow prerequisites contribute to the characteristics of flow experience. In this sense, the merging of action and awareness seems to result from the balance between challenge and skill, having clear goals in mind, being in control of the activity, and an interaction with time transformation. It is, however, beyond our reach to determine how time transformation and action/awareness merging influence one another. Still, time transformation has also been related to the sense of pleasure in the activity (autotelic experience) and being less aware of internal and external pressures (loss of self-consciousness).

One important characteristic of being in flow state is feeling “at one” with the activity (Jackson and Csikszentmihalyi, 1999). The dimensions and interactions presented above describe different aspects of being “at one” with the activity or fostering such feeling; however, action/awareness merging, loss of self-consciousness, concentration on task, and sense of control have all been criticized as being overlapping characteristics (Swann et al., 2018). While such criticism has its foundation, these dimensions appeared in our results as independent constructs interacting in a complex manner and representing distinct aspects of one mental state.

The dimensions of loss of self-consciousness and time transformation have been questioned regarding their applicability for athletes, as well as for tending to present low factor loadings for the general model of flow (Jackson and Eklund, 2002). These are the two least reported dimensions of flow in qualitative investigations with athletes (Swann et al., 2012); however, the low support for these dimensions in sports has been commonly overlooked (Swann et al., 2018). Present results have shown small contributions from both characteristics of flow along with inadequate internal consistency for time transformation (α = 0.68). These two dimensions had the lowest degrees of strength and closeness, with only time transformation showing some level of betweenness. Moreover, loss of self-consciousness was the only dimension negatively linked to performance. Still, these flow characteristics contributed to other aspects of the experience, such as the merging of action and awareness, which suggests that, even with an overall low support, there is relevant information within these dimensions’ items to be taken into consideration when studying flow.

### Limitations, Future Directions, and Practical Implications

As part of any investigation, a few limitations must be presented. Our sample was predominantly male (89%), undermining our understanding of flow and performance relationships for female athletes. Nonetheless, 87% of our target population (Brazilian competitors in the event) were male athletes. Another limitation is the assessment of only one sport, despite triathlon being composed of three different individual sports (long-distance running, cycling, and swimming); it does not mean that our results can be directly generalized to athletes from only one of these sports or even out of triathlon in general. Adopting a cross-sectional design using a memory-dependent subjective measurement of one’s propensity to experience flow is an important limiting factor as well.

Future studies seeking to better understand the relationship between flow and athletic performance could benefit from the use of both dispositional and state measurements of flow to analyze how general propensity to experience flow will contribute to event-specific experiences, and how both may influence performance. Researchers should use both subjective and objective indicators of performance, since flow is an individual mental state (subjective) happening in a competitive context where objective performance is the main determinant for winning. Including physiological data, such as VO_2__*max*_, will also benefit performance-related studies. Furthermore, the understanding of flow state specificities as a function of sex and type of sport (e.g., short-duration × long-duration; team sports × individual sports) also requires more attention. Adopting mixed methods to obtain and analyze quantitative and qualitative data is also advised to enrich the overall understanding of this complex phenomenon.

As practical implications, athletes, coaches, sports psychologists, and other professionals working with triathletes can better understand flow and its mechanisms within this sport, with the goal of increasing the frequency and intensity of this highly desirable mental state. In this sense, we observe that some dimensions of flow can be more directly focused, such as working with the athletes’ concentration and setting realistic, achievable, and motivating goals, while other dimensions seem to be a consequence of a variety of factors, such as the merging of action and awareness and the actual control over the activity being performed. Thus, directing attention toward aspects that can be more directly improved might, in turn, facilitate the occurrence of others. To develop balance between challenge and skill, the main condition for experiencing flow, we would like to highlight that both aspects go beyond improving one’s actual skill and choosing adequate challenges. Therefore, it is important to positively develop how athletes perceive their own skill level, as well as how they perceive and interpret contextual demands.

## Data Availability

All datasets generated for this study are included in the manuscript and/or the Supplementary Files.

## Ethics Statement

The present study was approved by the Ethics Committee of Universidade Estadual de Maringá, opinion number 2.287.443. The Brazilian Triathlon Confederation (CBTri) was contacted in order to obtain approval to collect data at the event. Athletes read and signed an informed consent in order to voluntarily accept participation. Only adults were recruited. No vulnerable population was involved.

## Author Contributions

WG and PA contributed to the conception or design of the study. MM, AC, and MR recruited and acquired data from the participants. JV and RC analyzed and interpreted the data. WG and RC wrote the manuscript. JN and LF critically revised the intellectual content.

## Conflict of Interest Statement

The authors declare that the research was conducted in the absence of any commercial or financial relationships that could be construed as a potential conflict of interest.
